# Tuberculosis Burden and Determinants of Treatment Outcomes According to Age in Brazil: A Nationwide Study of 896,314 Cases Reported Between 2010 and 2019

**DOI:** 10.3389/fmed.2021.706689

**Published:** 2021-07-27

**Authors:** Beatriz Barreto-Duarte, Mariana Araújo-Pereira, Betânia M. F. Nogueira, Luciana Sobral, Moreno M. S. Rodrigues, Artur T. L. Queiroz, Michael S. Rocha, Vanessa Nascimento, Alexandra B. Souza, Marcelo Cordeiro-Santos, Afrânio L. Kritski, Timothy R. Sterling, María B. Arriaga, Bruno B. Andrade

**Affiliations:** ^1^Laboratório de Inflamação e Biomarcadores, Instituto Gonçalo Moniz, Fundação Oswaldo Cruz, Salvador, Brazil; ^2^Multinational Organization Network Sponsoring Translational and Epidemiological Research Initiative, Salvador, Brazil; ^3^Curso de Medicina, Universidade Salvador, Laureate Universities, Salvador, Brazil; ^4^Programa de Pós-Graduação em Clínica Médica, Universidade Federal do Rio de Janeiro, Rio de Janeiro, Brazil; ^5^Faculdade de Medicina, Universidade Federal da Bahia, Salvador, Brazil; ^6^Instituto Brasileiro para Investigação da Tuberculose, Fundação José Silveira, Salvador, Brazil; ^7^Programa de Pós-graduação em Ciências da Saúde, Faculdade de Medicina da Bahia, Universidade Federal da Bahia, Salvador, Brazil; ^8^Curso de Medicina, Centro Universitário Faculdade de Tecnologia e Ciências, Salvador, Brazil; ^9^Escola Bahiana de Medicina e Saúde Pública, Salvador, Brazil; ^10^Laboratório de Análise e Visualização de Dados, Fundação Oswaldo Cruz, Porto Velho, Brazil; ^11^Center of Data and Knowledge Integration for Health, Instituto Gonçalo Moniz, Fundação Oswaldo Cruz, Salvador, Brazil; ^12^Fundação Medicina Tropical Doutor Heitor Vieira Dourado, Manaus, Brazil; ^13^Programa de Pós-Graduação em Medicina Tropical, Universidade do Estado do Amazonas, Manaus, Brazil; ^14^Faculdade de Medicina, Universidade Nilton Lins, Manaus, Brazil; ^15^Programa Acadêmico de Tuberculose da Faculdade de Medicina, Universidade Federal do Rio de Janeiro, Rio de Janeiro, Brazil; ^16^Division of Infectious Diseases, Department of Medicine, Vanderbilt University School of Medicine, Nashville, TN, United States

**Keywords:** tuberculosis, age, outcomes, pulmonary TB, extrapulmonary TB

## Abstract

Approximately 1.4 million people die annually worldwide from tuberculosis. Large epidemiologic studies can identify determinants of unfavorable clinical outcomes according to age, which can guide public health policy implementation and clinical management to improve outcomes. We obtained data from the national tuberculosis case registry; data were reported to the Brazilian National Program (SINAN) between 2010 and 2019. Clinical and epidemiologic variables were compared between age groups (child: <10 years, young: 10–24years, adult: 25–64years, and elderly: ≥65years). Univariate comparisons were performed together with second-generation *p*-values. We applied a backward stepwise multivariable logistic regression model to identify characteristics in each age group associated with unfavorable TB treatment outcomes. There were 896,314 tuberculosis cases reported during the period. Tuberculosis incidence was highest among adult males, but the young males presented the highest growth rate during the period. Directly observed therapy (DOT) was associated with protection against unfavorable outcomes in all age groups. The use of alcohol, illicit drugs, and smoking, as well as occurrence of comorbidities, were significantly different between age groups. Lack of DOT, previous tuberculosis, race, location of tuberculosis disease, and HIV infection were independent risk factors for unfavorable outcome depending on the age group. The clinical and epidemiological risk factors for unfavorable tuberculosis treatment outcomes varied according to age in Brazil. DOT was associated with improved outcomes in all age groups. Incidence according to age and sex identified adults and young males as the groups that need prevention efforts. This supports implementation of DOT in all populations to improve tuberculosis outcomes.

## Introduction

Tuberculosis (TB) remains a major public health problem worldwide, accounting for 10 million new cases and 1.4 million deaths in 2019 according to the World Health Organization (WHO) (1). Brazil is among the 22 high TB burden countries identified by the WHO, which account for 82% of TB cases worldwide (1). Previous studies have assessed the dynamics of age on TB epidemiology in different settings around the world (2, 3) and also in Brazil (4). Recently, an investigation of a national database identified important associations between adolescence and TB notification over several years in South Africa (5). Additional studies using nationwide datasets in TB-endemic countries can help delineate strategies focused on age groups to identify interventions that could improve outcomes.

The Brazilian National Plan to Control Tuberculosis (PNCT) was implemented in 1999. Following WHO recommendations, the program set goals of a cure rate >85% and loss to follow-up <5% (6). In addition, the PNCT employed in 2003 directly observed therapy (DOT), which is a tool proposed by the WHO for supervising and documenting the intake of anti-TB medication (7), to reduce the incidence of TB drug resistance and increase the likelihood of successful treatment outcomes. However, in non-research settings, DOT has not been uniformly implemented among all persons treated for TB in Brazil. TB control at the population level requires a better understanding of the clinical and epidemiological characteristics of the people affected by TB. This was facilitated by the Information System for Notifiable Diseases (SINAN), a health information platform of mandatory notification and continuous update, created in 1993 and maintained by the Ministry of Health of Brazil (8). SINAN collects standardized data, from diagnosis to treatment outcome from all Brazilian states, to inform and guide health professionals and policymakers about TB epidemiology in Brazil, as well as measuring the impact of the policies implemented (6).

Through the SINAN database, there is valuable information for TB control in Brazil, such as clinical and demographic characteristics, proven risk factors for TB treatment outcomes, and the actual outcomes. Moreover, it is feasible to analyze such characteristics over time, enabling the understanding of TB epidemiology nationwide over several years. In the present study, SINAN data from a 10-year period (2010–2019) were analyzed to assess temporal changes in incidence, TB clinical manifestations, and clinical outcomes, in individuals stratified by age group and biological sex.

## Methods

### Ethics Statement

All data available for the years 2010–2019 were obtained from the government platform (publicly available) pre-processed by the Ministry of Health, which included verification of duplicate registration, consistency, and completeness of registered data (8), following the regulations of Resolution No. 466/12 on Research Ethics of the National Health Council, Brazil. This study was exempt from Institutional Review Board (IRB) approval by the IRB at Fundação Oswaldo Cruz, Salvador.

### Overall Study Design

We performed an observational study comparing the baseline clinical and sociodemographic characteristics and outcome determinants according to four age groups, which were defined as follows: children (0–9.9 years) (9); young (10–24.9 years) (10); adults (25–64.9 years); and elderly (≥65 years) following a previously published guideline (11). The primary hypothesis was that the general characteristics of TB patients registered in the SINAN database as well as the main risk factors for unfavorable treatment outcomes varied according to the age of the patients.

### Data Collection

The present study obtained data from SINAN (access date 9/22/2020), available on the website (8) and implemented, supported, and maintained by the Brazilian Ministry of Health. SINAN monitors information on more than 40 reportable diseases, including TB (12). Between January 2010 and December 2019, there were 896,314 TB cases reported to SINAN, diagnosed by bacteriology/positive culture, chest radiography or histopathology or clinical criteria, in the case of extrapulmonary TB, according to Brazilian TB guidelines (6). In addition to the type of TB diagnosis, SINAN notification forms also collected data on individual characteristics, including clinical (HIV, diabetes, smear, and culture among others) and sociodemographic (sex, age, ethnicity, and literacy) data. All variables used in this study are shown in detail in Table 1.

**Table 1:** Population characteristics by year.

	**2010**	**2011**	**2012**	**2013**	**2014**	**2015**	**2016**	**2017**	**2018**	**2019**	
**Characteristics**	***N* = 87,171**	***N* = 89,735**	***N* = 88,229**	***N* = 88,339**	***N* = 86,967**	***N* = 87,105**	***N* = 87,820**	***N* = 82,303**	***N* = 96,292**	***N* = 92,353**	**χ^**2**^trend *p*-value**
Female, *n* (%)	28,867 (33.1)	29,407 (32.8)	28,734 (32.6)	28,788 (32.6)	27,824 (32.0)	27,109 (31.1)	27,202 (31.0)	27,734 (30.0)	29,195 (30.3)	27,909 (30.2)	0.46
Age group, *n* (%)											0.827
Child	1,594 (1.82)	1,641 (1.83)	1,572 (1.78)	1,618 (1.85)	1,373 (1.57)	1,324 (1.52)	1,461 (1.68)	1,558 (1.78)	1,755 (2.01)	1,770 (2.03)	
Young	14,894 (17.08)	15,454 (17.22)	15,391 (17.4)	15,195 (17.20)	14,871 (17.06)	15,442 (17.71)	15,822 (18.15)	17,127 (19.64)	18,838 (21.61)	19,555 (22.43)	
Adult	62,576 (71.79)	64,279 (71.63)	63,196 (71.63)	63,135 (71.47)	62,522 (71.72)	61,884 (70.99)	61,841 (70.9)	64,296 (73.76)	66,190 (75.93)	62,292 (71.45)	
Elderly	7,766 (8.9)	8,062 (8.98)	7,752 (8.78)	8,005 (9.06)	7,818 (8.97)	8,114 (9.3)	8,309 (9.53)	8,948 (10.3)	9,099 (10.4)	8,239 (9.45)	
Ethnicity, *n* (%)											0.108
Asian	755 (0.87)	729 (0.81)	729 (0.83)	681 (0.77)	624 (0.72)	592 (0.68)	580 (0.66)	715 (0.77)	729 (0.76)	698 (0.76)	
Black	11,681 (13.4)	12,169 (13.6)	11,985 (13.6)	11,407 (12.9)	11,345 (13.0)	11,041 (12.7)	11,182 (12.7)	11,552 (12.5)	12,364 (12.8)	11,922 (12.9)	
Indigenous	889 (1.02)	1,009 (1.12)	898 (1.02)	958 (1.08)	893 (1.03)	1,017 (1.17)	989 (1.13)	930 (1.01)	951 (0.99)	930 (1.01)	
Pardo	35,898 (41.2)	38,310 (42.7)	38,575 (43.7)	39,433 (44.6)	39,221 (45.1)	40,155 (46.1)	41,137 (46.8)	44,548 (48.3)	47,118 (48.9)	45,354 (49.1)	
White	29,960 (34.4)	30,438 (33.9)	29,616 (33.6)	28,866 (32.7)	27,792 (32.0)	27,346 (31.4)	26,887 (30.6)	27,583 (29.9)	28,387 (29.5)	26,243 (28.4)	
Literate, *n* (%)	38,459 (44.1)	49,055 (54.7)	48,724 (55.2)	48,702 (55.1)	47,978 (55.2)	48,241 (55.4)	48,781 (55.5)	51,194 (55.5)	54,922 (57.0)	51,851 (56.1)	0.16
HIV infection, *n* (%)	9,659 (11.1)	10,080 (11.2)	10,089 (11.4)	10,174 (11.5)	10,383 (11.9)	10,097 (11.6)	9,736 (11.1)	10,119 (11.0)	9,970 (10.4)	9,124 (9.88)	**<0.001**
ART, *n* (%)	23 (0.24)	21 (0.24)	32 (0.32)	128 (1.26)	953 (9.18)	2,102 (20.81)	3,292 (33.81)	3,967 (39.2)	4,020 (40.32)	3,211 (35.19)	**<0.001**
Alcohol consumption, *n* (%)	12,851 (14.7)	13,853 (15.4)	13,953 (15.8)	14,097 (16.0)	14,176 (16.3)	15,174 (17.4)	15,568 (17.7)	16,858 (18.3)	18,227 (18.9)	17,015 (18.4)	0.958
Illicit drug use, *n* (%)	334 (0.38)	2,098 (2.34)	2,687 (3.05)	3,269 (3.70)	5,579 (6.42)	10,342 (11.9)	11,423 (13.0)	13,467 (14.6)	15,165 (15.7)	14,522 (15.7)	**<0.001**
Smoking habits, *n* (%)	106 (0.12)	240 (0.27)	1,457 (1.65)	2,096 (2.37)	5,597 (6.44)	15,826 (18.2)	18,826 (21.4)	21,328 (23.1)	23,560 (24.5)	22,490 (24.4)	**<0.001**
Diabetes *n* (%)	5,101 (5.85)	5,593 (6.23)	5,727 (6.49)	5,922 (6.70)	5,707 (6.56)	6,117 (7.02)	6,385 (7.27)	6,785 (7.35)	7,356 (7.64)	7,318 (7.92)	0.447
Smear positive, *n* (%)	47,715 (54.9)	48,957 (56.5)	47,961 (56.2)	47,134 (53.4)	46,739 (53.8)	45,971 (53.9)	45,501 (53.8)	45,290 (51.1)	46,153 (50.0)	42,985 (48.8)	0.169
Culture positive, *n* (%)	11,387 (13.1)	12,321 (13.7)	12,968 (14.7)	13,859 (15.7)	14,972 (17.2)	18,020 (20.7)	18,358 (20.9)	20,367 (22.1)	21,267 (22.1)	15,656 (17.0)	**0.03**
Abnormal X-ray, *n* (%)	69,168 (79.3)	71,150 (79.3)	68,990 (78.2)	68,053 (77.0)	65,748 (75.6)	63,258 (72.6)	63,371 (72.2)	64,885 (70.3)	67,911 (70.5)	64,515 (69.9)	**0.01**
TB Status, *n* (%)											0.399
New case	72,151 (82.8)	74,144 (82.6)	72,552 (82.2)	72,476 (82.0)	70,951 (81.6)	70,300 (80.7)	70,631 (80.4)	73,970 (80.1)	77,091 (80.1)	73,906 (80.0)	
Prior TB	14,687 (16.8)	15,330 (17.1)	15,354 (17.4)	15,550 (17.6)	15,729 (18.1)	16,518 (19.0)	16,874 (19.2)	17,996 (19.5)	18,874 (19.6)	18,004 (19.5)	
Treatment, *n* (%)											**<0.001** ^**a**^ **, 0.015** ^**b**^
Received DOT	36,870 (42.3)	40,673 (45.3)	41,717 (47.3)	40,771 (46.2)	37,291 (42.9)	29,423 (33.8)	30,736 (35.0)	32,899 (35.6)	34,594 (35.9)	22,364 (24.2)	
No received DOT	38,266 (43.9)	37,247 (41.5)	34,682 (39.3)	38,073 (43.1)	35,459 (40.8)	30,986 (35.6)	32,387 (36.9)	33,580 (36.4)	33,868 (35.2)	26,953 (29.2)	
Type of TB, *n* (%)											0.684
EPTB	11,580 (13.3)	12,058 (13.4)	11,906 (13.5)	11,893 (13.5)	11,172 (12.8)	10,747 (12.3)	11,096 (12.6)	11,477 (12.4)	12,177 (12.6)	11,804 (12.8)	
PTB	72,567 (83.2)	74,650 (83.2)	73,181 (82.9)	73,431 (83.1)	72,871 (83.8)	73,549 (84.4)	74,023 (84.3)	78,103 (84.6)	81,120 (84.2)	77,914 (84.4)	
PTB and EPTB	2,996 (3.44)	3,005 (3.35)	3,123 (3.54)	2,889 (3.27)	2,854 (3.28)	2,764 (3.17)	2,665 (3.03)	2,674 (2.90)	2,958 (3.07)	2,568 (2.78)	
Comorbidity, *n* (%)											0.56
Cancer	805 (0.92)	802 (0.89)	877 (0.99)	758 (0.86)	744 (0.86)	755 (0.87)	787 (0.90)	770 (0.83)	851 (0.88)	770 (0.83)	
COPD	161 (0.18)	165 (0.18)	197 (0.22)	109 (0.12)	109 (0.13)	112 (0.13)	117 (0.13)	125 (0.14)	153 (0.16)	143 (0.15)	
Hypertension	7,885 (9.05)	8,301 (9.25)	8,091 (9.17)	8,576 (9.71)	8,573 (9.86)	8,796 (10.1)	9,030 (10.3)	9,706 (10.5)	9,867 (10.2)	9,269 (10.0)	
Renal disease	78 (0.09)	80 (0.09)	77 (0.09)	69 (0.08)	80 (0.09)	59 (0.07)	73 (0.08)	74 (0.08)	60 (0.06)	62 (0.07)	
Others	11,850 (13.6)	12,640 (14.1)	12,529 (14.2)	11,944 (13.5)	11,710 (13.5)	12,359 (14.2)	12,543 (14.3)	13,621 (14.8)	13,844 (14.4)	14,201 (15.4)	
No condition	66,390 (76.2)	67,745 (75.5)	66,451 (75.3)	66,878 (75.7)	65,750 (75.6)	65,017 (74.6)	65,270 (74.3)	68,007 (73.7)	71,517 (74.3)	67,904 (73.5)	
Outcome description, *n* (%)											**<0.001**^**a**^, 0.366^b^
Cure	59,870 (68.7)	62,367 (69.5)	59,817 (67.8)	60,694 (68.7)	59,521 (68.4)	57,977 (66.6)	59,388 (67.6)	61,493 (66.6)	60,981 (63.3)	18,516 (20.0)	
Death	6,635 (7.61)	6,686 (7.45)	6,560 (7.44)	6,702 (7.59)	6,833 (7.86)	7,019 (8.06)	6,881 (7.84)	7,152 (7.75)	7,003 (7.27)	4,917 (5.32)	
Failure	668 (0.77)	681 (0.76)	694 (0.79)	735 (0.83)	1,070 (1.23)	1,473 (1.69)	1,480 (1.69)	1,587 (1.72)	1,683 (1.75)	1,120 (1.21)	
Loss follow up	10,643 (12.2)	10,683 (11.9)	11,017 (12.5)	11,653 (13.2)	11,395 (13.1)	10,731 (12.3)	10,991 (12.5)	11,644 (12.6)	12,021 (12.5)	4,841 (5.24)	
Relapse	1,860 (2.13)	1,992 (2.22)	2,116 (2.40)	2,193 (2.48)	1,755 (2.02)	1,668 (1.91)	1,872 (2.13)	2,063 (2.24)	2,075 (2.15)	1,361 (1.47)	
Transferred out	5,804 (6.66)	5,954 (6.64)	5,932 (6.72)	4,708 (5.33)	4,837 (5.56)	4,905 (5.63)	4,759 (5.42)	5,194 (5.63)	6,202 (6.44)	7,138 (7.73)	
Outcome, *n* (%)											**0.012**^**a**^, 0.694^b^
Unfavorable	25,610 (30.0)	25,996 (29.4)	26,319 (30.6)	25,991 (30.0)	25,890 (30.3)	25,796 (30.8)	25,983 (30.4)	27,640 (31.0)	28,984 (32.2)	19,377 (51.1)	
Favorable	59,870 (70.0)	62,367 (70.6)	59,817 (69.4)	60,694 (70.0)	59,521 (69.7)	57,977 (69.2)	59,388 (69.6)	61,493 (69.0)	60,981 (67.8)	18,516 (48.9)	

*Bold font indicates statistical significance. Data are shown as number and frequency (percentage). Data were compared between years using the Pearson's χ^2^trend test. Age Groups: Children (0–9.9 years); young (10–24.9 years); adults (25–64.9 years); elderly (≥65 years)*.

a *Comparison between 2010 and 2010*.

b *Comparisons between 2010 and 2018*.

*DOT, directly observed treatment; COPD, chronic obstructive pulmonary disease; TB, Tuberculosis; PTB, Pulmonary Tuberculosis; EPTB, Extrapulmonary Tuberculosis*.

### Outcome Definition

For this study, a favorable treatment outcome was defined as cure or completed treatment; unfavorable outcome was defined as treatment failure, lost to follow-up, relapse, or death during treatment. The definitions for clinical and bacteriological cure, failure, lost to follow-up and death for both cohorts corresponded with those in the Manual of Recommendations for the Control of TB of Brazil (6). The outcomes “unknown,” “ongoing treatment,” and “transferred out” were not considered for the univariate and multivariate analysis of the TB treatment outcomes. The outcome definitions are shown in Supplementary Table 1.

### Statistical Analysis

The TB notification rate was determined by dividing the annual number of notified TB patients by the estimated mid–year population (according to data from the Instituto Brasileiro de Geografia e Estatística, IBGE) (13) multiplied by 100,000. The annual mid–year population estimate was derived from the 2010 national census and adjusted for annual population growth assuming linear growth between census estimates. Quantitative variables were presented as medians and interquartile range (IQR), while categorical variables were presented as percentages (%). Categorical and trend analysis for social and clinical characteristics were assessed using the Chi–squared test (χ^2^) and χ^2^ test for trend), except for age and TB rates, for which the Mann–Kendall (MK) test was used to identify differences among years. During review of the data, some inconsistencies were observed, such as children from 0 to 4 years old with habits of consumption of tobacco, alcohol, and illegal drugs. Due to the lack of a way to confirm these data, consumption habits of these substances were excluded from the analysis in this age group.

To perform a backward stepwise logistic regression, all clinical and epidemiological parameters were included in the univariate analyses and were included in the multivariable models to evaluate unfavorable outcome of the treatment. *P* < 0.05 were considered statistically significant. In addition to the *p*-value, the second-generation *p*-value (*p*δ-value) and the delta-gap (Δ) (when applicable) were calculated to evaluate the outcomes (8). For interpretation, we considered all cases in which *p*δ = 0 were clinically of interest, and statistically significant; when *p*δ = 1, the data affirmed only the effects that were null or almost null and that had little clinical interest, which would confirm the lack of association. When *p*δ = 0.5, then the data were inconclusive. In case of *p*δ = 0, the delta-gap (Δ) was calculated, which is defined as the distance between the intervals in δ units. The delta-gap value was directly related with the difference in distribution of values between the groups; the higher the delta value, the greater the effect size (14).

## Results

### Population Characteristics Over Time

**Figure 1:**
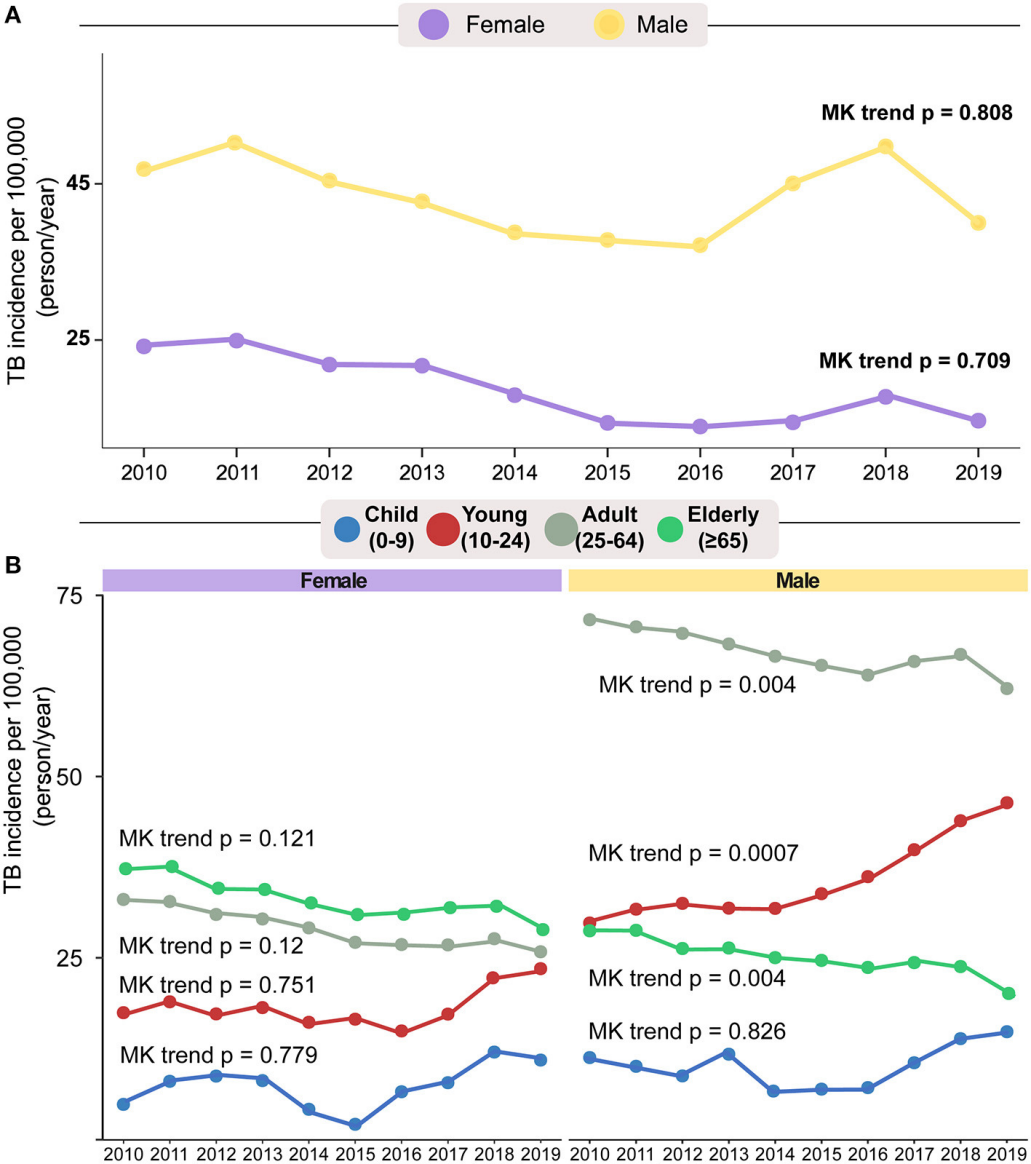
General population TB rates by sex **(A)** and age group **(B)** between 2010 and 2019. Mann–Kendal trend test was used to calculate changes in incidence among years. According to sex and/or age group, on panel **(A)**, it is possible to observe that there was a small decrease in the incidence of women with tuberculosis (purple). In age stratification **(B)**, only the incidence in children has not changed over the years. Age Groups: Children (0–9.9 years); young (10–24.9 years); Adults (25–64.9 years); Elderly (≥65 years).

Overall, there were 896,314 TB cases reported in Brazil between 2010 and 2019. Most TB patients were male (66.9–70%) and the age group most affected was 25–64 years (70.9–75.93%). Most of the patients self-reported their race as *pardo* (41.2–49.1%) and 44.1–56.1% were literate. The percentage of patients co-infected with HIV declined over time (χ^2^trend *p* < 0.001), being significantly lower in 2019. Over time, the frequency of patients who reported alcohol consumption, of those diagnosed with DM, and with smear-positive TB remained similar (Table 1). The proportion of *Mycobacterium tuberculosis* (Mtb) positive cultures increased over time (13.1–22.1, χ^2^trend *p* = 0.03) while abnormal findings on chest x-rays became less frequent (χ^2^trend *p* = 0.01; Table 1). The incidence of TB remained stable over time in men (MK-trend *p* = 0.808) and women (MK-trend *p* = 0.709; Figure 1A). However, within each sex, changes in TB incidence according to age group differed over the study period (Figure 1B). The incidence in adult (MK-trend *p* = 0.12), elderly (MK-trend *p* = 0.121), young (MK-trend *p* = 0.751) and child females (MK-trend *p* = 0.779) remained stable. In young men, TB incidence increased significantly over the study period (MK-trend *p* = 0.0007), while the incidence in elderly (MK-trend *p* = 0.004) and adult men (MK-trend *p* = 0.004) decreased. The TB incidence in male children did not change significantly (Figure 1B).

In all the years evaluated, the proportion of cases that were new exceeded 80% (80–82.8%), while the proportion treated with DOT decreased over time (χ^2^trend *p* < 0.001), even when we did not take 2019 into account (whose patients were still being treated, χ^2^trend *p* = 0.015; Table 1). The most frequent form of TB was pulmonary (PTB) (82.9–84.6%), followed by extrapulmonary TB (EPTB) (12.3–13.5%) and, finally, combined PTB-EPTB disease (2.78–3.54%). The most frequent comorbidity in all years was hypertension (9–10.5%), followed by DM (5.85–7.92%). Between 2010 and 2018 the cure rate of patients undergoing TB treatment was over 60% (63.3–68.7%), while the death rate ranged between 7.61 and 7.27%. Many patients diagnosed in 2019 were likely still in treatment when data were collected, and therefore there were missing values for this variable. Of note, we found a large number of missing and poorly filled variables in the data, shown in detail in Supplementary Table 2.

### Analysis by Age Group

**Figure 2:**
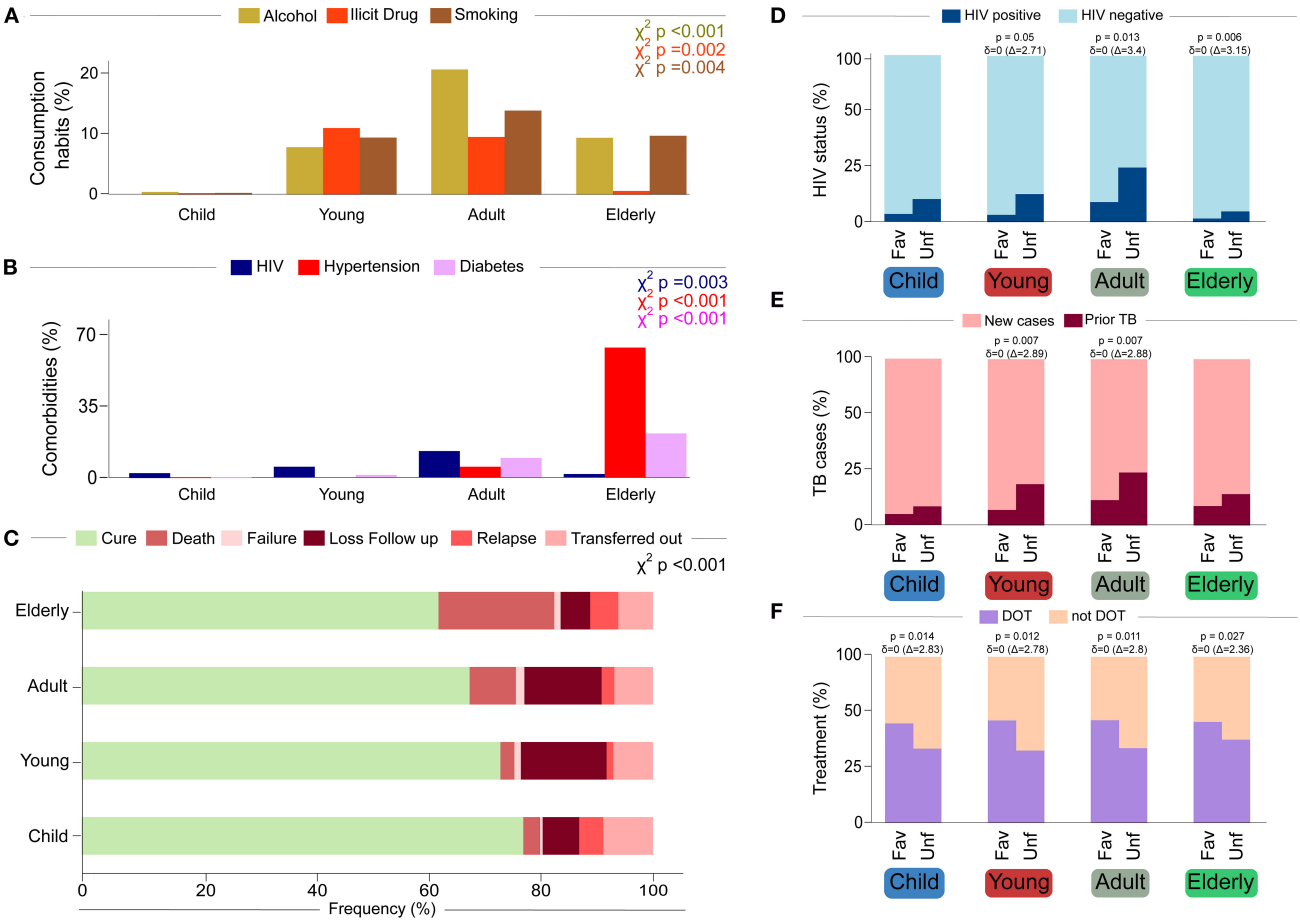
Characteristics of each age group **(A–C)** and outcome category **(D–F)**. **(A)** Consumption habits, we observed that young people and adults have greater consumption habits of alcohol, cigarettes, and illegal drugs than children and the elderly. **(B)** Comorbidities, while adults have a higher prevalence of HIV. The elderly has a higher prevalence of diabetes and hypertension. **(C)** Outcome description. The frequency of favorable outcome (cure) decreases according to age, being higher in children and lower in the elderly. **(D)** HIV status. Positive serology for HIV infection is correlated with an unfavorable treatment outcome in young, adults, and elderly. **(E)** TB Status. Relapse cases of TB are correlated with an unfavorable treatment outcome in young and adults. **(F)** Observed Treatment. In all age groups, receive a DOT is correlated with a favorable treatment outcome. Age Groups: Children (0–9.9 years); young (10–24.9 years); Adults (25–64.9 years); Elderly(≥65 years).

Approximately 70% (*n* = 632,211) of TB diagnoses in Brazil during the study period were made in people 25–64 years old (Table 2). This was the age group that had the highest proportion of people reporting at least one previous episode of TB (20.5%, χ^2^trend *p* < 0.008) among the four age groups evaluated in this study, as well as a higher proportion of persons living with HIV (PLWH) (13.9%, χ^2^trend *p* < 0.003), alcohol consumption (20.7%, χ^2^trend *p* < 0.001), and smoking (13.9%, χ^2^trend *p* < 0.001; Figure 2A). As expected, the frequency of literate people was significantly different between subgroups, given that the first subgroup consisted of children mainly of preschool age (χ^2^trend *p* < 0.008; Table 2). Children also had the lowest percentage of PTB (68.3%, χ^2^trend *p* < 0.004) and the highest proportion of EPTB among the age groups of the study (27.3%; Supplementary Figure 1). Consistent with this result, children also had a higher percentage of chest X-rays considered clinically normal (14.8%, χ^2^trend *p* = 0.012), in addition to a higher frequency of negative sputum smears (19.1%, χ^2^trend *p* < 0.001), negative TB culture (6.87%, χ^2^trend *p* < 0.001) and lower detection of DM (0.31%, χ^2^trend *p* < 0.001, Figure 2B). Children also had a higher proportion of cure after treatment (69.22%, χ^2^trend *p* < 0.001, Figure 2C). Young people more frequently reported illicit drug use (11%, χ^2^trend *p* < 0.001, Figure 2A), while elderly individuals were more likely diagnosed with DM (16.7%, χ^2^trend p < 0.001), hypertension (65.6%, χ^2^trend *p* < 0.001) and also had a higher mortality rate (18.6%, χ^2^trend *p* < 0.001; Figures 2B,C).

**Table 2:** Population characteristics by age categories.

	**Child**	**Young**	**Adult**	**Elderly**	
**Characteristics**	***N* = 15,666**	***N* = 162,589**	***N* = 632,211**	***N* = 82,112**	**p-value**
Female, *n* (%)	7,434 (47.5)	56,772 (34.9)	187,995 (29.7)	29,269 (35.6)	0.063
Ethnicity, *n* (%)					0.793
Asian	78 (0.50)	1,252 (0.77)	4,548 (0.72)	924 (1.13)	
Black	1,412 (9.01)	20,271 (12.5)	86,369 (13.7)	8,173 (9.95)	
Indigenous	1,099 (7.02)	2,101 (1.29)	4,952 (0.78)	1,181 (1.44)	
Pardo	7,180 (45.8)	78,417 (48.2)	288,677 (45.7)	33,797 (41.2)	
White	4,566 (29.1)	48,387 (29.8)	197,042 (31.2)	32,087 (39.1)	
Literate, *n* (%)	2,199 (14.0)	111,724 (68.7)	347,495 (55.0)	26,372 (32.1)	**<0.001**
HIV infection, *n* (%)	556 (3.55)	8,976 (5.52)	87,800 (13.9)	1,804 (2.20)	**0.002**
ART, *n* (%)	108 (0.69)	2,245 (1.38)	14,933 (2.36)	413 (0.50)	0.624
Alcohol consumption, *n* (%)	0 (0.00)	12,728 (7.83)	130,850 (20.7)	7,726 (9.41)	**<0.001**
Illicit drug use, *n* (%)	0 (0.00)	17,962 (11.0)	60,237 (9.53)	493 (0.60)	**0.002**
Smoking habits, *n* (%)	0 (0.00)	15,320 (9.42)	87,890 (13.9)	7,988 (9.73)	**0.003**
Diabetes, *n* (%)	49 (0.31)	1,563 (0.96)	46,480 (7.35)	13,742 (16.7)	**<0.001**
Smear positive, *n* (%)	1,557 (10.7)	91,707 (57.8)	333,002 (54.0)	36,669 (45.9)	**<0.001**
Culture positive, *n* (%)	656 (4.19)	30,014 (18.5)	117,133 (18.5)	10,992 (13.4)	**0.008**
Abnormal X-ray, *n* (%)	11,096 (70.8)	119,658 (73.6)	469,800 (74.3)	63,793 (77.7)	0.739
TB Status, *n* (%)					0.09
New case	14,378 (91.8)	140,149 (86.2)	500,527 (79.2)	69,958 (85.2)	
Prior TB	1,227 (7.83)	22,057 (13.6)	129,414 (20.5)	11,672 (14.2)	
Supervised treatment, *n* (%)					0.958
Received DOT	5,839 (37.3)	65,867 (40.5)	243,834 (38.6)	30,481 (37.1)	
No received DOT	5,492 (35.1)	61,134 (37.6)	242,838 (38.4)	30,681 (37.4)	
Type of TB, *n* (%)					**0.004**
EPTB	4,276 (27.3)	18,174 (11.2)	82,180 (13.0)	10,712 (13.0)	
PTB	10,700 (68.3)	140,432 (86.4)	528,018 (83.5)	69,258 (84.3)	
PTB+EPTB	688 (4.39)	3,904 (2.40)	21,659 (3.43)	2,083 (2.54)	
Comorbidity, *n* (%)					**<0.001**
Cancer	33 (0.21)	948 (0.58)	5,059 (0.80)	1,854 (2.26)	
COPD	6 (0.04)	25 (0.02)	492 (0.08)	868 (1.06)	
Hypertension	0 (0.00)	0 (0.00)	34,245 (5.42)	53,849 (65.6)	
Renal disease	1 (0.01)	80 (0.05)	295 (0.05)	336 (0.41)	
Others	2,103 (13.4)	79,727 (49.0)	41,834 (6.62)	3,485 (4.24)	
No condition	13,523 (86.3)	81,798 (50.3)	550,279 (87.0)	21,712 (26.4)	
Outcome description, *n* (%)					0.343
Cure	10,842 (69.2)	107,159 (65.9)	393,360 (62.2)	47,141 (57.4)	
Death	412 (2.63)	3,542 (2.18)	46,871 (7.41)	15,256 (18.6)	
Failure	60 (0.38)	1,652 (1.02)	8,619 (1.36)	832 (1.01)	
Loss follow up	899 (5.74)	21,985 (13.5)	78,524 (12.4)	3,932 (4.79)	
Relapse	591 (3.77)	1,708 (1.05)	12,801 (2.02)	3,718 (4.53)	
Transferred out	1,220 (7.79)	10,143 (6.24)	39,130 (6.19)	4,558 (5.55)	
Outcome, *n* (%)					0.113
Unfavorable	3,182 (22.7)	39,030 (26.7)	185,945 (32.1)	28,296 (37.5)	
Favorable	10,842 (77.3)	107,159 (73.3)	393,360 (67.9)	47,141 (62.5)	

*Bold font indicates statistical significance. Data are shown as number and frequency (percentage). Data were compared between years using the Pearson's χ^2^trend test. Age Groups: Children (0–9.9 years); young (10–24.9 years); adults (25–64.9 years); elderly (≥65 years)*.

*DOT, directly observed treatment; COPD, chronic obstructive pulmonary disease; TB, Tuberculosis; PTB, Pulmonary Tuberculosis; EPTB, Extrapulmonary Tuberculosis*.

*Others comorbidities: It did not include hypertension, kidney disease, cancer, and COPD*.

### Anti-TB Treatment Outcomes Over Time

Over time, the proportion of treatment outcomes did not differ substantially (Table 1), but there was some variability according to age group (Figure 2C, χ^2^trend *p* < 0.001). Younger patients had a higher proportion of cure. Among unfavorable outcomes (non-cure), loss to follow-up was more frequent in young and adults, while death was more frequent in the elderly (Table 2 and Figure 2C). These outcomes were grouped as favorable and unfavorable (Table 3 and Supplementary Figure 2). Except for children, the other groups of patients with non-cure had a higher percentage of HIV co-infection (Figure 2D). The most affected group was adults (*p* = 0.013, *p*δ = 0), followed by young (*p* = 0.05, *p*δ = 0) and elderly people (*p* = 0.006, *p*δ = 0; Figure 2D). Adults and young people with an unfavorable outcome also had higher frequencies of prior TB cases (Figure 2E), both with *p* = 0.007 and *p*δ = 0. In all age groups, the use of DOT was more frequent in patients with a favorable outcome (Figure 2F).

**Table 3:** Population characteristics by categorical outcome.

	**All**	**Unfavorable**	**Favorable**		
**Characteristics**	***N* = 818,210**	***N* = 257586**	***N* = 560624**	***p*-value**	***p*δ-value**
Female, *n* (%)	259,110 (31.7)	72269 (28.1)	186841 (33.3)		1
Age group, *n* (%)				0.241	0.5
Child	14,024 (1.71)	3,182 (1.23)	10,842 (1.93)		
Young	146,189 (17.87)	39,030 (12.15)	107,159 (19.11)		
Adult	559,305 (68.35)	185,945 (72.18)	373,360 (66.6)		
Elderly	75,437 (9.22)	28,296 (10.9)	47,141 (8.4)		
Ethnicity *n* (%)				0.593	1
Asian	6,223 (0.76)	1,802 (0.70)	4,421 (0.79)		
Black	106,135 (13.0)	37,494 (14.6)	68,641 (12.2)		
Indigenous	8,708 (1.06)	2,149 (0.83)	6,559 (1.17)		
Pardo	371,627 (45.4)	120,177 (46.7)	251,450 (44.9)		
White	262,253 (32.1)	74,681 (29.0)	187,572 (33.5)		
Literate, *n* (%)	445,662 (54.5)	125,377 (48.7)	320,285 (57.1)	0.294	0 (Δ = 0.14)
HIV infection, *n* (%)	91,882 (11.2)	49,642 (19.3)	42,240 (7.53)	**0.004**	0 (Δ = 1.38)
ART, *n* (%)	15,527 (16.9)	7,650 (15.41)	7,877 (18.64)	0.89	1
Alcohol consumption, *n* (%)	131,687 (16.0)	57,421 (22.3)	74,266 (13.2)	**0.065**	0 (Δ = 0.67)
Illicit drug use, *n* (%)	68,557 (8.37)	32,511 (12.6)	36,046 (6.42)	0.3	0 (Δ = 0.08)
Smoking habits, *n* (%)	88,922 (10.87)	34,522 (13.4)	54,396 (9.7)	0.449	1
Diabetes, *n* (%)	56,282 (6.88)	16,557 (6.43)	39,725 (7.09)	0.786	1
Smear positive, *n* (%)	428,296 (53.6)	123,759 (49.3)	304,537 (55.6)	0.453	1
Culture positive, *n* (%)	148,436 (18.1)	44,187 (17.2)	104,249 (18.6)	0.748	1
Abnormal X-ray, *n* (%)	612,756 (74.9)	194,509 (75.5)	418,247 (74.6)	1	1
TB Status, *n* (%)				**0.014**	0 (Δ = 1.02)
New case	665,236 (81.3)	181,561 (70.5)	483,675 (86.3)		
Prior TB	150,310 (18.4)	74,248 (28.8)	76,062 (13.6)		
Treatment, *n* (%)				**0.012**	0 (Δ = 0.96)
Received DOT	333,100 (40.7)	73,073 (28.4)	260,027 (46.4)		
No received DOT	320,530 (39.2)	108,547 (42.1)	211,983 (37.8)		
Type of TB				0.697	1
EPTB	105,523 (12.9)	31,243 (12.1)	74,280 (13.2)		
PTB	686,346 (83.9)	214,434 (83.2)	471,912 (84.2)		
PTB and EPTB	26,234 (3.21)	11,812 (4.59)	14,422 (2.57)		
Comorbidity, *n* (%)				0.88	1
Cancer	7,320 (0.89)	2,565 (1.00)	4,755 (0.85)		
COPD	1,301 (0.16)	640 (0.25)	661 (0.12)		
Hypertension	80,806 (9.88)	27,707 (10.8)	53,099 (9.47)		
Kidney disease	659 (0.08)	371 (0.14)	288 (0.05)		
Others	115,928 (14.2)	33,592 (13.0)	82,336 (14.7)		
No condition	612,173 (74.8)	192,703 (74.8)	419,470 (74.8)		

*Bold font indicates statistical significance. Data are shown as number and frequency (percentage). Data were compared between years using the Pearson's χ^2^trend test. Age Groups: Children (0–9.9 years); young (10–24.9 years); adults (25–64.9 years); elderly (≥65 years)*.

*DOT, directly observed treatment; COPD, chronic obstructive pulmonary disease; TB, Tuberculosis; PTB, Pulmonary Tuberculosis; EPTB, Extrapulmonary Tuberculosis. Other comorbidities: did not include hypertension, kidney disease, cancer, and COPD*.

### TB in Children

**Figure 3:**
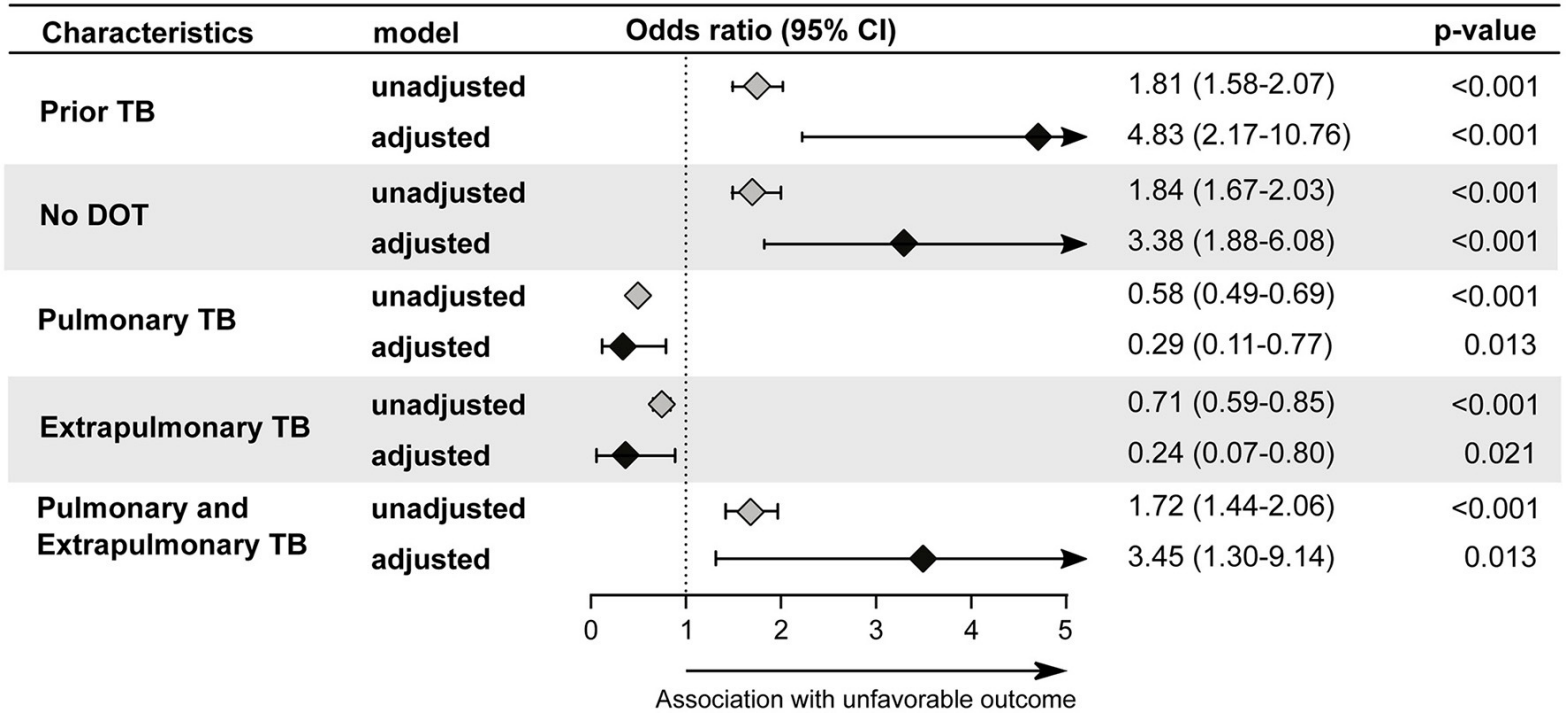
Backward stepwise logistic regression model test independent associations between all the relevant clinical and epidemiological parameters and treatment outcome in children (0–9 years). The unfavorable outcome was used as reference to test associations. Only parameters which remained with *p* < 0.05 in in the adjusted model (95%CI: 95% confidence interval) were plotted. Adjustment was performed for each parameter: race (reference: indigenous); male (reference: female); illiterate(reference: literate); prior TB (reference: new case); no DOT (reference: Received DOT).Pulmonary TB (reference: Pulmonary and Extrapulmonary TB); Extrapulmonary TB (Reference: Pulmonary and Extrapulmonary TB); Pulmonary and Extrapulmonary TB (reference: Pulmonary TB); HIV infection (reference: without HIV infection); Alcohol Consumption (reference: no alcohol consumption); Diabetes (reference: no diabetes); Illicit drug use (reference: no illicit drug use); Smoking habit (reference: no smoking). Cancer (reference: no condition); COPD (reference: no condition); Kidney disease (reference: no condition); Hypertension (reference: no condition); Other comorbidities (reference: no condition); Abnormal chest X-ray (reference: normal chest X-ray). TB, tuberculosis; DOT, directly observed treatment; COPD, chronic obstructive pulmonary disease.

Children diagnosed with TB between the years 2010 and 2019 maintained a similiar proportion of sex distribution over the years (Table 1), as well as the incidence per 100 thousand inhabitants, did not change significantly (Figure 1B). As in other age groups, the most common form of TB in children was PTB, but the frequency of EPTB was relatively high, representing 27.3% of cases (Supplementary Figure 1). Children also had a low smear-positive rate (35.8%) being diagnosed by this method (Supplementary Figure 1). A binomial logistic regression analysis was performed to test independent associations between the parameters analyzed and treatment outcomes in children (Figure 3). We found that the unfavorable outcomes were increased in patients with prior TB (adjusted odds ratio [aOR]: 4.83, 95% confidence interval [CI]: 2.17–10.76, *p* < 0.001), in those who did not undergo DOT (aOR: 3.38, 95% CI: 1.88–6.08, *p* < 0.001) and in those who presented with simultaneously PTB-EPTB (aOR: 3.45, 95% CI: 1.3–9.14, *p* = 0.013).

### TB in the Young

**Figure 4:**
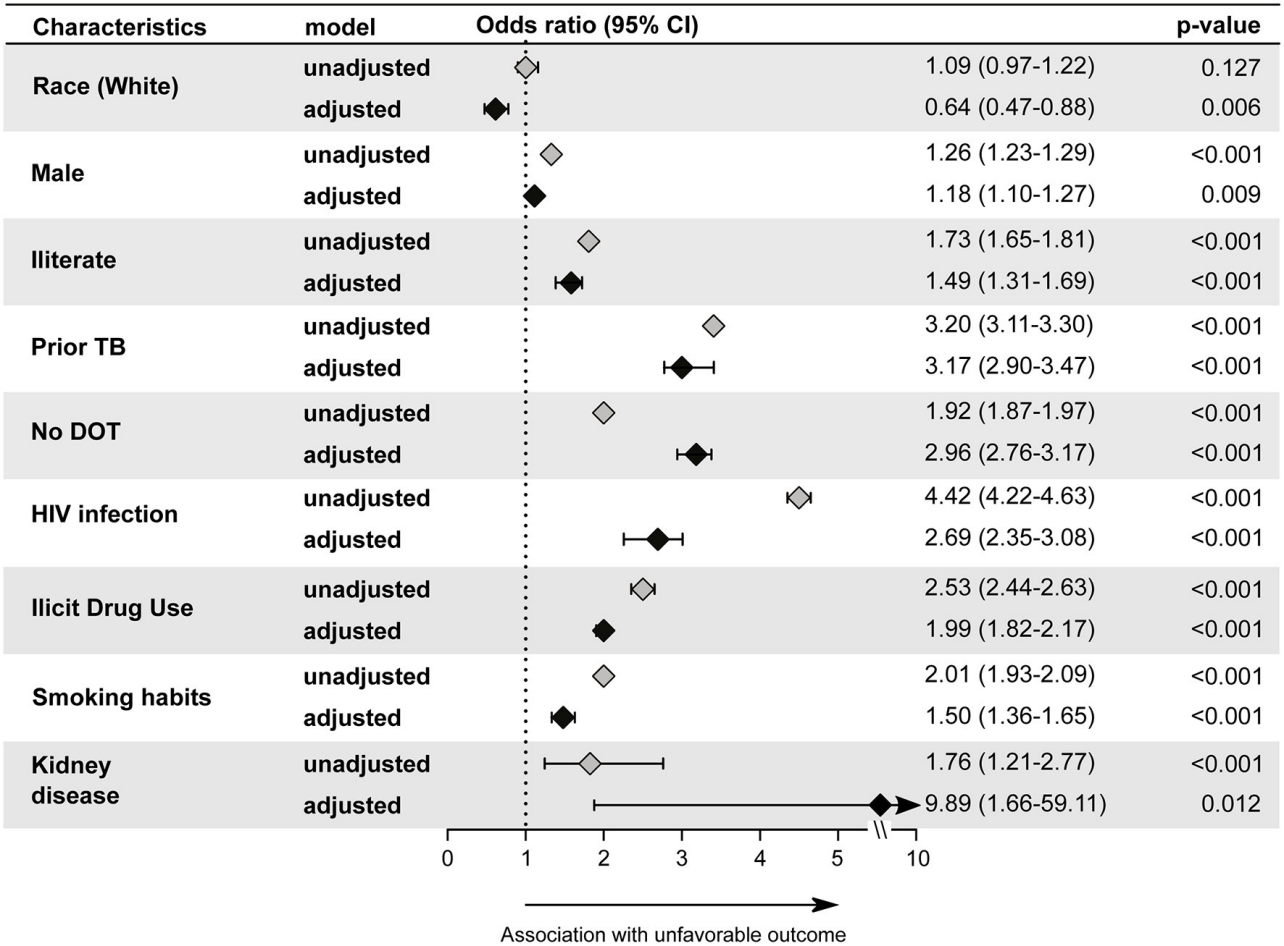
Backward stepwise logistic regression model test independent associations between all the relevant clinical and epidemiological parameters and treatment outcome in young group (10–24 years). The unfavorable outcome was used as reference to test associations. Only parameters which remained with *p* < 0.05 in in the adjusted model (95%CI: 95% confidence interval) were plotted. Adjustment was performed for each parameter: race (reference: indigenous); male (reference: female); illiterate (reference: literate); prior TB (reference: new case); no DOT (reference: Received DOT). Pulmonary TB (reference: Pulmonary and Extrapulmonary TB); Extrapulmonary TB (Reference: Pulmonary and Extrapulmonary TB); Pulmonary and Extrapulmonary TB (reference: Pulmonary TB); HIV infection (reference: without HIV infection); Alcohol consumption (reference: no alcohol consumption); Diabetes (reference: no diabetes); Illicit drug use (reference: no illicit drug use); Smoking habit (reference: no smoking). Cancer (reference: no condition); COPD (reference: no condition); Kidney disease (reference: no condition); Hypertension (reference: no condition); Other comorbidities (reference: no condition); Abnormal chest X-ray (reference: normal chest X-ray). TB, tuberculosis; DOT, directly observed treatment; COPD, chronic obstructive pulmonary disease; Other comorbidities, did not include HAS, kidney disease, cancer, and COPD.

Young patients diagnosed with TB between 2010 and 2019 showed a significant change (χ^2^trend *p*-value = 0.048) in the distribution by sex over the years (Table 1 and Figure 1B). This change was characterized by a significant increase in TB incidence in men over the study period (MK-trend *p* = 0.0007), while the TB incidence in women remained relatively stable (MK-trend *p* = 0.751; Figure 1B). The most common (86.4%) form of TB in young patients was PTB, (Supplementary Figure 1) and a high positive smear rate was observed (76.9%, Supplementary Figure 1). Binomial logistic regression analysis showed results similar those in children, with prior TB (aOR: 3.17, 95%CI: 2.90–3.47, *p* < 0.001) and no DOT indication (aOR: 2.96, 95% CI: 2.76–3.17, *p* < 0.001) independently associated with unfavorable treatment outcome (Figure 4). Additional factors associated with unfavorable outcomes were male (aOR: 1.18, 95%CI: 1.10–1.27, *p* < 0.001), illiteracy (aOR: 1.49, 95%CI: 1.31–1.69, *p* < 0.001), HIV infection (aOR: 2.69, 95%CI: 2.35–3.08, *p* < 0.001), illicit drug use (aOR: 1.99, 95%CI: 1.82–2.17, *p* < 0.001), smoking (aOR: 1.50, 95% CI: 1.36–1.65, *p* < 0.001), and kidney disease (aOR: 9.89, 95%CI: 1.66–59.11, *p* = 0.012).

### TB in Adults

**Figure 5:**
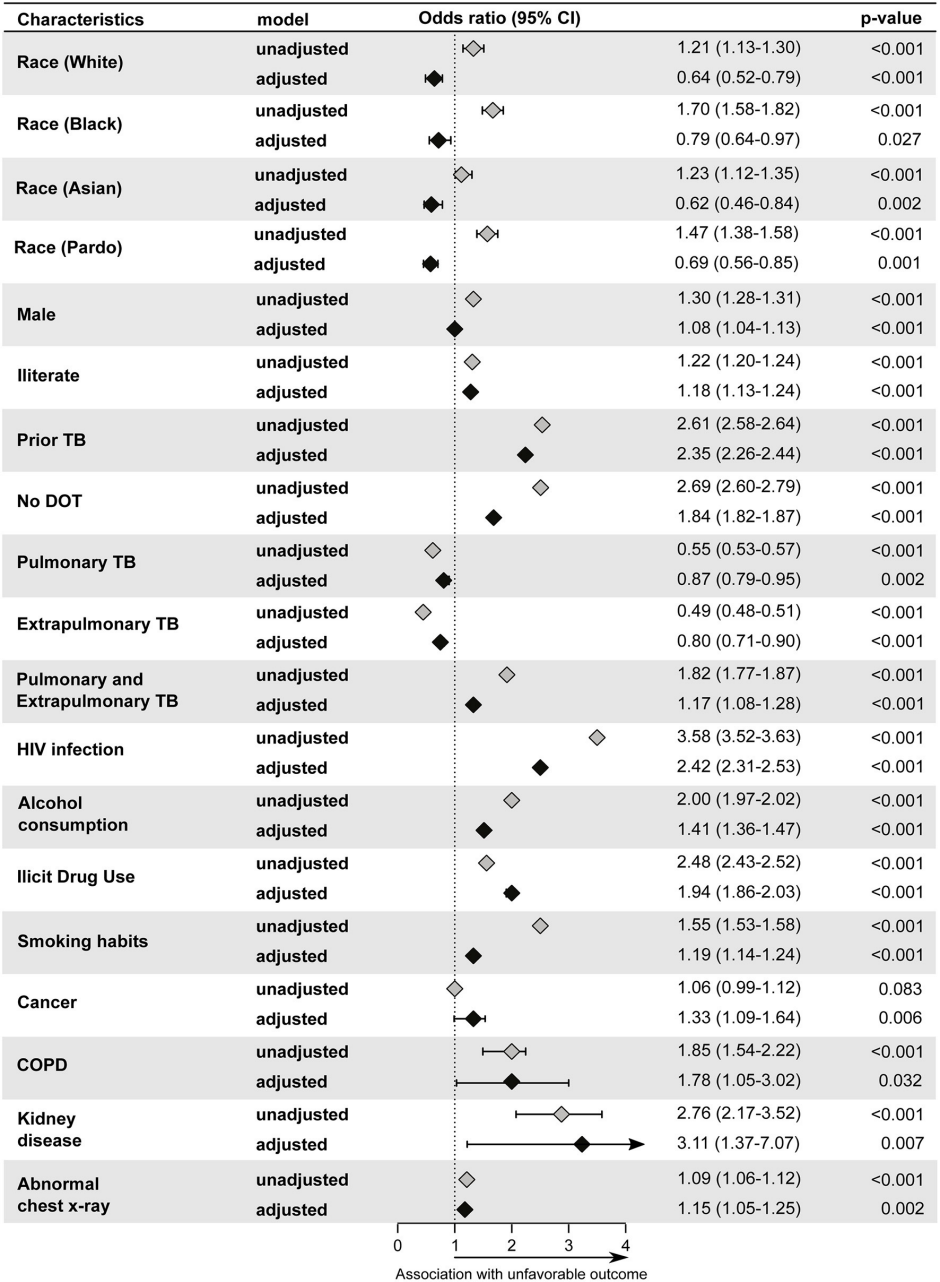
Backward stepwise logistic regression model test independent associations between all the relevant clinical and epidemiological parameters and treatment outcome in adult group (25–64). The unfavorable outcome was used as reference to test associations. Only parameters which remained with *p* < 0.05 in in the adjusted model (95%CI: 95% confidence interval) were plotted. Adjustment was performed for each parameter: race (reference: indigenous); male (reference: female); illiterate(reference: literate); prior TB (reference: new case); no DOT (reference: DOT indication).Pulmonary TB (reference: Pulmonary and Extrapulmonary TB); Extrapulmonary TB (Reference: Pulmonary and Extrapulmonary TB); Pulmonary and Extrapulmonary TB (reference: Pulmonary TB); HIV infection (reference: without HIV infection); Alcohol Consumption (reference: no alcohol consumption); Diabetes (reference: no diabetes); Illicit drug use (reference: no illicit drug use); Smoking habit (reference: no smoking). Cancer (reference: no condition); COPD (reference: no condition); Kidney disease (reference: no condition); Hypertension (reference: no condition); Other comorbidities (reference: no condition); Abnormal chest X-ray (reference: normal chest X-ray). TB, tuberculosis; DOT, directly observed treatment; COPD, chronic obstructive pulmonary disease.

Adults diagnosed with TB showed a similar proportion of men and women between 2010 to 2019 (Table 1), but in terms of incidence, there was a significant decrease only in male sex (women MK-trend *p* = 0.12; men MK-trend *p* = 0.004) during the 10-year study period (Figure 1B). Similar to the rest of the population, the most common type of TB was pulmonary (86.5%, Supplementary Figure 1). In adults, the proportion of positive sputum was slightly lower than that found in young people, corresponding to 70.5% of the TB adult population (Supplementary Figure 1). The binomial logistic regression analysis showed similar results to children and young patients, with prior TB (aOR: 2.35, 95% CI: 2.26–2.44, *p* < 0.001) and no DOT (aOR: 2.29, 95% CI: 2.60–2.79, *p* < 0.001) independently associated with unfavorable outcome (Figure 5). As seen among children, the presence of both PTB-EPTB was associated with unfavorable outcomes (aOR: 1.17, 95%CI: 1.07–1.28, *p* < 0.001) and as in young people, the following factors were also associated with unfavorable outcomes: HIV infection (aOR: 2.42, 95%CI: 2.31–2.53, *p* < 0.001), illicit drug use (aOR: 1.94, 95%CI: 1.86–2.03, *p* < 0.001) smoking (aOR: 1.19, 95%CI: 1.14–1.24, *p* < 0.001), and kidney disease (aOR: 3.11, 95%CI: 1.37–7.07, *p* = 0.007). Being male (aOR: 1.08, 95%CI: 1.04–1.13, *p* < 0.001), white (aOR: 0.64, 95%CI: 0.53–0.77, *p* < 0.001) black (aOR: 0.79, 95%CI: 0.64–0.97, *p* = 0.027), Asian (aOR: 0.62, 95%CI: 0.46–0.84, *p* = 0.002), *pardo* (aOR: 0.69, 95%CI: 0.56–0.85, *p* = 0.001), alcohol consumption (aOR: 1.41, 95%CI: 1.36–1.47, *p* < 0.001), illiteracy (aOR: 1.18, 95%CI: 1.13–1.24, *p* < 0.001), abnormal chest X-ray (aOR: 1.15, 95%CI: 1.05–1.15, *p* = 0.002), cancer (aOR: 1.33, 95%CI: 1.09–1.64, *p* = 0.006) or COPD (aOR: 1.78, 95%CI: 1.05–3.02, *p* = 0.032) were also significantly associated in this subpopulation.

### TB in Elderly

**Figure 6:**
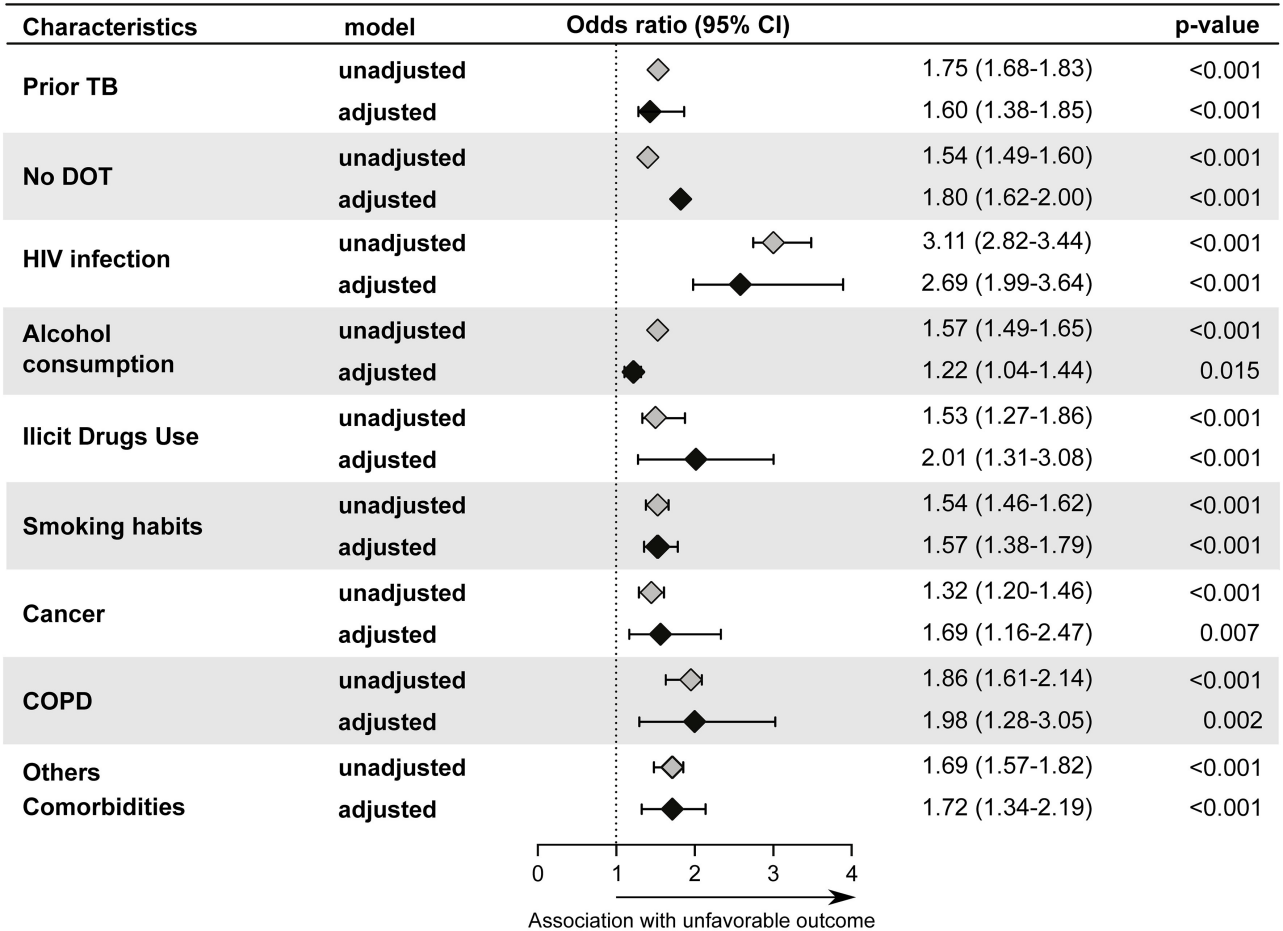
Backward stepwise logistic regression model test independent associations between all the relevant clinical and epidemiological parameters and treatment outcome in elderly group (≥65 years). The unfavorable outcome was used as reference to test associations. Only parameters which remained with *p* < 0.05 in in the adjusted model (95%CI: 95% confidence interval) were plotted. Adjustment was performed for each parameter: race (reference: indigenous); male (reference: female); illiterate(reference: literate); prior TB (reference: new case); no DOT (reference: DOT indication); Pulmonary TB (reference: Pulmonary and Extrapulmonary TB); Extrapulmonary TB (Reference: Pulmonary and Extrapulmonary TB); Pulmonary and Extrapulmonary TB (reference: Pulmonary TB); HIV infection (reference: without HIV infection); Alcohol Consumption (reference: no alcohol consumption); Diabetes (reference: no diabetes); Illicit drug use (reference: no illicit drug use); Smoking habit (reference: no smoking). Cancer (reference: no condition); COPD (reference: no condition); Kidney disease (reference: no condition); Hypertension (reference: no condition); Other comorbidities (reference: no condition); Abnormal chest X-ray (reference: normal chest X-ray). TB, tuberculosis; DOT, directly observed treatment; COPD, chronic obstructive pulmonary disease; Other comorbidities: It did not include HAS, kidney disease, cancer, and COPD.

The changes concerning biological sex affected by TB in the elderly population between the years 2010 and 2019 showed a pattern similar to that found in the adult population, with little change in frequencies over the years (Table 1) but showed a significant decrease only in male sex with regard to TB incidence per 100 thousand population (women MK-trend *p* = 0.121; men MK-trend *p* = 0.004, Figure 1B). The frequency of PTB in this population was very close to that found in young people and adults (84.4%, Supplementary Figure 1), but the proportion with smear-positive disease was considerably lower, at 62% (Supplementary Figure 1). Unlike other sub-populations, ethnicity was not associated with unfavorable outcome in elderly (Figure 6). However, factors such as prior TB (aOR: 1.60, 95%CI: 1.38–1.85, *p* < 0.001), HIV infection (aOR: 2.69, 95%CI: 1.99–3.64, *p* < 0.001), alcohol consumption (aOR: 1.22, 95%CI: 1.04–1.44, *p* = 0.015), illicit drugs use (aOR: 2.01, 95%CI: 1.31–3.08, *p* < 0.001) and smoking (aOR: 1.57, 95%CI: 1.38–1.79, *p* < 0.001), in addition to cancer (aOR: 1.69, 95%CI: 1.69–2.47, *p* = 0.007), COPD (aOR: 1.98, 95%CI: 1.28–3.05, *p* = 0.002), other comorbidities (aOR: 1.72, 95%CI: 1.34–2.19, *p* < 0.001) and no DOT (aOR: 1.80, 95%CI: 1.62–2.00, *p* < 0.001) were associated with unfavorable outcome.

## Discussion

In the present study, we investigated the epidemiologic characteristics of TB in the Brazilian population between 2010 and 2019 through data from SINAN. We assessed favorable and unfavorable outcomes as well as the factors associated with each. We focused on identifying the specific risk factors for unfavorable outcomes in each age group, aiming to provide more detailed information for targeted interventions in each group. Our results highlight the importance of DOT for success of TB treatment and encourages the amplification of this strategy in the country, as has been recommend by WHO since 1993. DOT was associated with a considerable increase in favorable outcomes in all studied age groups.

We evaluated the population characteristics of TB cases over a 10-year period. The TB incidence rate in Brazil remained high, and of the cases reported between 2010 and 2019 most were male (2:1), adults, and self-reported as *pardo*. This profile is similar to that observed in previous years in Brazil (15). The decrease in the incidence reported between the years 2011–2016 suggests that there was a positive impact of the expansion of public policies leading to an economic incentive, since patients who received cash transfer from governmental programs were about 7% more likely to have a favorable TB treatment outcome (16). In addition, the increase in incidence recorded between the years 2017–2018 corresponds to the end of the implementation of GeneXpert, resulting in a greater use of this as a TB diagnosis strategy, which was between 2013 and 2017, thus being an effective strategy for the optimizing diagnosis even when sputum smear is negative.

As also found in previous years, substance use (alcohol, tobacco, and illicit drugs) was commonly reported in the studied population, but there was a significant increase throughout the years. The increase in the prevalence of tobacco smoking in this population is a surprising finding, as it is in the opposite direction of the general population in Brazil (17). Conversely, illicit drug use seemed to follow the national trend of increasing over the years. Although there is no information about which specific drugs were responsible for our findings, national surveys have demonstrated an increase in the use of marijuana and cocaine in the Brazilian population in the past decade. The use of these drugs is particularly prevalent and growing in young males, which is compatible with the findings of the present study (18–20). This is an important finding in the young population, particularly since we also found that substance use was significantly associated with unfavorable TB treatment outcomes. Moreover, previous studies have shown that the use of these substances are also risk factors for developing TB (17, 21, 22).

Another interesting finding was that the prevalence of DM in our population was similar to the general population (23). Although there has been an increase in DM prevalence in Brazil in recent years, the increase was not statistically significant in our study population (24, 25). One of the reasons for the relatively low prevalence of DM in our study could be the fact that many cases of DM reported in SINAN are self-reported. A systematic review of DM prevalence in Brazil has shown that studies that used complex diagnoses (self-reported and laboratory investigation) of DM have found a much higher prevalence than studies that relied solely on self-report. Of note, Brazilian TB treatment guidelines suggest but do not mandate DM testing in TB patients (as with HIV testing is, following WHO recommendation) (6). Emphasis on the importance of detecting DM is important to reduce the underdiagnosis of DM.

The proportion of TB cases with positive cultures increased over time. This may be due to greater access to the test and to technical improvements. As an example, a study performed in a state reference laboratory found an increase of 61.5% in the positive results for Mtb after the implementation of a semi-automated procedure (26). It is possible that health professionals may be more aware of the importance of performing cultures for all TB cases. Another hypothesis pertains to the recommendation to perform universal culture and DST on all presumed TB cases, made by WHO and followed by the Brazilian MoH after 2015. It is worth noting that, despite the improvement, the access to Mtb culture in Brazil is still far from ideal. In 2019, only 24% of patients with new cases of TB had cultures performed (12), which likely means that many patients treated for TB did not have TB. As expected, our analysis found that the number of positive cultures in children was low compared to the other age groups, which is likely due to the difficulty in collecting sputum (27), and that children often have paucibacillary disease, with a higher percentage of nodular lesions and fewer cavitary lung lesions. Another important observation was that children had a higher frequency of EPTB and a higher frequency of both PTB and EPTB, possibly due to immunological immaturity, which can contribute to the hematogenous spread of the disease in children.

TB is one of the most common opportunistic infections in persons living with HIV (PLWH) and the main cause of death in this population (28). The prevalence of HIV/TB co-infection has declined between 2010 and 2019 worldwide, with rates of co-infection decreasing from 1.7 million to about 900 thousand people (1). In Brazil, the Ministry of Health (MoH) has reported an increase in HIV diagnosis, but a reduction in the number of AIDS cases and deaths related to HIV. Our study also found a significant reduction in the cases of HIV/TB co-infection, but HIV infection was significantly associated with a greater number of unfavorable outcomes in all age groups, except for children.

The decline of HIV/TB follows the reduction in AIDS cases in Brazil, which is a reflection of the expansion in HIV diagnosis, HIV prevention efforts, as well as access to treatment. Since 2013, ART has been offered to all PLWH, regardless of CD4 count (29).In 2017, Brazil began providing dolutegravir (DTG), a very effective antiretroviral with fewer side effects, as part of the first line scheme. As a result, in 2018, 86% of PLWH knew their status, 67% were on ART and 60% were virally suppressed (29). The reduction in HIV/TB cases and the higher proportion of people on ART are important factors explaining the decrease in TB mortality rate noted in our analysis and reinforces the essential role of HIV care policies in the control of TB.

Interestingly there was no difference in treatment outcomes among the age groups, but there were risk factors for unfavorable outcomes specific for each age group; knowing them is essential to guide public health interventions (4). For example, it was noted in our analysis that, in disagreement with all the other curves evaluated by age that showed a reduction or stabilization, the number of young men with TB significantly increased over recent years. This subpopulation has a social risk behavior associated with a higher prevalence of illicit drug use, and once diagnosed with TB, young people have difficulty staying in care. Thus, the young age group is an important target for public health measures and knowing detailed information about them is key.

Finally, we have evaluated patient characteristics according to treatment outcomes. Along the years studied, there was an increase in unfavorable outcomes, mainly caused by higher rates of treatment failure. After adjustment for confounders alcohol consumption, illicit drug use, tobacco smoking, HIV infection, kidney disease, prior TB, and having PTB-EPTB were associated with unfavorable outcomes. First, our results highlight the already known strong connection of TB and social factors, such as race and substance use (30, 31). It is not within the scope of this work to detail the reasons why these characteristics affect TB outcome, but factors such as adherence to therapy, access to care and time to diagnosis may be related and need to be further investigated. Comorbidities like HIV (32) and kidney disease have previously been shown to be associated with worse outcomes (33, 34). Surprisingly, DM was not found to affect treatment outcomes, and our hypothesis for this was discussed earlier. Having PTB-EPTB and previous episodes have previously been shown to be associated with worse outcomes (35). The first may be due to the greater difficulty of treatment (35) as well as disease severity, while the second could be due to patient non-adherence, or an increased risk of drug resistance in TB relapse cases.

This study had some limitations. It is unclear whether reporting of information was uniform throughout the country. There was a high level of under-reporting for several variables in the questionnaire. In addition, when assessing patient outcomes, 2019 was incomplete since some patients were still in follow-up. It is important to know that, with exception of name, ethnicity, age, HIV-infection, and more “clinical” variables, other fields such as consumption habits and literacy presented a considerable number of missing data.

With the above limitations noted, a study of this magnitude is extremely important to provide a national view of TB in Brazil over the past decade. This allowed us to investigate not only how public policies applied during that time period influenced the rates and treatment outcomes of TB patients in the country, but also to identify interventions that might improve TB treatment outcomes. Increased implementation of DOT in Brazil across all age groups would likely improve TB treatment outcomes in the country.

## Data Availability Statement

The datasets presented in this study can be found in online repositories. The names of the repository/repositories and accession number(s) can be found below: the data was extracted from the public database: DataSUS (TABNET)—http://tabnet.datasus.gov.br (in Brazilian Portuguese).

## Ethics Statement

The studies involving human participants were reviewed and approved by Comitê de Ética em Pesquisa, Instituto Gonçalo Moniz, Fundação Oswaldo Cruz, Salvador, Bahia, Brazil. Written informed consent from the participants' legal guardian/next of kin was not required to participate in this study in accordance with the national legislation and the institutional requirements.

## Author Contributions

BB-D, MA-P, MA, LS, MSR, VN, AS, and BA contributed to conception and design of the study. BB-D, MA-P, MA, MMSR, AQ, TS, and BA performed the data curation. BB-D, MA-P, and MA processed and analyzed the data, and worked on data visualization. BB-D, MA-P, MA, BN, TS, and BA wrote the first draft of the manuscript. MC-S and AK revised and contributed to the structuring of the article. TS and BA supervised the research. All authors contributed to manuscript revision, read, and approved the submitted version of the manuscript.

## Conflict of Interest

The authors declare that the research was conducted in the absence of any commercial or financial relationships that could be construed as a potential conflict of interest.

## Publisher's Note

All claims expressed in this article are solely those of the authors and do not necessarily represent those of their affiliated organizations, or those of the publisher, the editors and the reviewers. Any product that may be evaluated in this article, or claim that may be made by its manufacturer, is not guaranteed or endorsed by the publisher.
