# Supplementary material for: Tuberculosis Burden and Determinants of Treatment Outcomes According to Age in Brazil: A Nationwide Study of 896,314 Cases Reported Between 2010 and 2019
Source: Front Med (Lausanne). 2021 Jul 27;8:706689. doi: 10.3389/fmed.2021.706689 (PMC8354381; doi:10.3389/fmed.2021.706689)
Supplement: Supplementary file 1 [file Data_Sheet_1.pdf]

Tuberculosis Burden and Determinants of Treatment Outcomes According to Age in Brazil: A Nationwide Study of 896,314 Cases Reported Between 2010 and 2019

Beatriz Barreto-Duarte, Mariana Araújo-Pereira, Betânia M. F. Nogueira, Luciana Sobral, Moreno M. S. Rodrigues, Artur T. L. Queiroz, Michael S. Rocha, Vanessa Nascimento, Alexandra B. Souza, Marcelo Cordeiro-Santos, Afrânio L. Kritski, Timothy R. Sterling, María B. Arriaga, Bruno B. Andrade

Supplementary Material

Content

1. Supplementary Table 1
2. Supplementary Table 2
3. Supplementary Figure 1
4. Supplementary Figure 2

Supplementary Tables:

Supplementary Table 1. Definition of tuberculosis treatment outcomes

Type of outcome treatment	Outcome tuberculosis treatment	SINAN[a]
Favorable	**Cure**	When pulmonary TB patients, initially sputum smear or Xpert positive, present, during treatment, with at least two negative sputum smears: one in the follow-up phase and the other at the end of treatment.
	**Completed treatment**	Completion of treatment based on clinical criteria and radiological: (i) when the patient has not undergone sputum examination due to absence sputum and are discharged based on clinical data and complementary exams, (ii) in cases of initially negative pulmonary tuberculosis; (iii) in cases of extrapulmonary tuberculosis
Unfavorable	**Loss to follow up**	A patient who has failed to attend the unit for more than 30 consecutive days after the expected return date. In supervised treatment cases, the period of 30 days was from the last date of taking the drug.
	**Relapse**	When the individual who presented active tuberculosis and was previously treated and cured presents the disease again, regardless of the time since the previous treatment.
	**Death**	On the occasion of knowledge of the patient's death during treatment.
	**Failure**	When sputum positivity persists at the end treatment. Also classified as failure are patients who beginning of treatment are strongly positive (++ or +++) and maintain this until the fourth month, or those with initial positivity followed by negativity and new positivity for two consecutive months, from the fourth month of treatment.
	**Transferred out**	When the patient is transferred to another health service.

Table note: [a]Described in the Manual of Recommendations for the Control of TB of Brazil. Abbreviations: SINAN: Sistema de Informação de Agravos de Notificação. TB: Tuberculosis.

Supplementary Table 2. Frequency of missing values in each analyzed parameter

Characteristics	All N=896314	2010 N=87171	2011 N=89735	2012 N=88229	2013 N=88339	2014 N=86967	2015 N=87105	2016 N=87820	2017 N=92303	2018 N=96292	2019 N=92353
Gender missing (%)	0 (0)	0 (0)	0 (0)	0 (0)	0 (0)	0 (0)	0 (0)	0 (0)	0 (0)	0 (0)	0 (0)
Ethnicity missing (%)	70503 (7.8)	7988 (9.16)	7080 (7.89)	6426 (7.28)	6994 (7.92)	7092 (8.15)	6954 (7.98)	7045 (8.02)	6975 (7.56)	6743 (7.00)	7206 (7.80)
Literate missing (%)	294472 (32.8)	36841 (42.3)	27071 (30.2)	26795 (30.4)	27534 (31.2)	27645 (31.8)	27690 (31.8)	28354 (32.3)	30515 (33.1)	30975 (32.2)	31052 (33.6)
HIV infection missing (%)	229995 (25.6)	32781 (37.6)	30836 (34.4)	28511 (32.3)	24846 (28.1)	21580 (24.8)	18205 (20.9)	17316 (19.7)	17232 (18.7)	17107 (17.8)	21581 (23.4)
ART missing (%)	0 (0)	0 (0)	0 (0)	0 (0)	0 (0)	0 (0)	0 (0)	0 (0)	0 (0)	0 (0)	0 (0)
Alcohol consumption missing (%)	74065 (8.3)	9258 (10.6)	9156 (10.2)	8452 (9.58)	7876 (8.92)	7717 (8.87)	6691 (7.68)	6136 (6.99)	5967 (6.46)	5966 (6.20)	6846 (7.41)
Illicit drug use missing (%)	372676 (41.6)	84861 (97.4)	69553 (77.5)	68169 (77.3)	65255 (73.9)	45612 (52.4)	9289 (10.7)	7576 (8.63)	7264 (7.87)	7272 (7.55)	7825 (8.47)
Smoking habits missing (%)	370656 (41.3)	84865 (97.4)	69580 (77.5)	68209 (77.3)	65341 (74.0)	45806 (52.7)	8807 (10.1)	7106 (8.09)	6873 (7.45)	6605 (6.86)	7464 (8.08)
Diabetes missing (%)	78525 (8.8)	10147 (11.6)	9810 (10.9)	9053 (10.3)	8497 (9.62)	8298 (9.54)	7072 (8.12)	6408 (7.30)	6281 (6.80)	6061 (6.29)	6898 (7.47)
Smear missing (%)	210702 (23.5)	17287 (19.8)	16213 (18.1)	16410 (18.6)	18649 (21.1)	18825 (21.6)	21104 (24.2)	20543 (23.4)	24684 (26.7)	28282 (29.4)	28705 (31.1)
Culture missing (%)	655644 (73.1)	68422 (78.5)	69169 (77.1)	67604 (76.6)	66333 (75.1)	63791 (73.4)	60523 (69.5)	60788 (69.2)	62750 (68)	65638 (68.2)	70626 (76.5)
Abnormal X-ray missing (%)	181891 (20.3)	13256 (15.2)	13631 (15.2)	14347 (16.3)	15279 (17.3)	16649 (19.1)	19632 (22.5)	20095 (22.9)	22701 (24.6)	23414 (24.3)	22887 (24.8)
TB Status missing (%)	3226 (0.36)	333 (0.38)	261 (0.29)	323 (0.37)	313 (0.35)	287 (0.33)	287 (0.33)	315 (0.36)	337 (0.37)	327 (0.34)	443 (0.48)
Supervised treatment missing (%)	207475 (23.1)	12035 (13.8)	11815 (13.2)	11830 (13.4)	9495 (10.7)	14217 (16.3)	26696 (30.6)	24697 (28.1)	25824 (28.0)	27830 (28.9)	43036 (46.6)
Type of TB missing (%)	499 (0.06)	28 (0.03)	22 (0.02)	19 (0.02)	126 (0.14)	70 (0.08)	45 (0.05)	36 (0.04)	49 (0.05)	37 (0.04)	67 (0.07)
Comorbidity missing (%)	28 (0.003)	2 (0.00)	2 (0.00)	7 (0.01)	5 (0.01)	1 (0.00)	7 (0.01)	0 (0.00)	0 (0.00)	0 (0.00)	4 (0.00)
Outcome missing (%)	78104 (8.7)	1691 (1.94)	1372 (1.53)	2093 (2.37)	1654 (1.87)	1556 (1.79)	3332 (3.83)	2449 (2.79)	3170 (3.43)	6327 (6.57)	54460 (59.0)

Table note: Missing data are shown as number and frequency (percentage). Abbreviations: ART: antiretroviral treatment; DOT: directly observed treatment; TB: Tuberculosis;

Supplementary Figures and Legends:

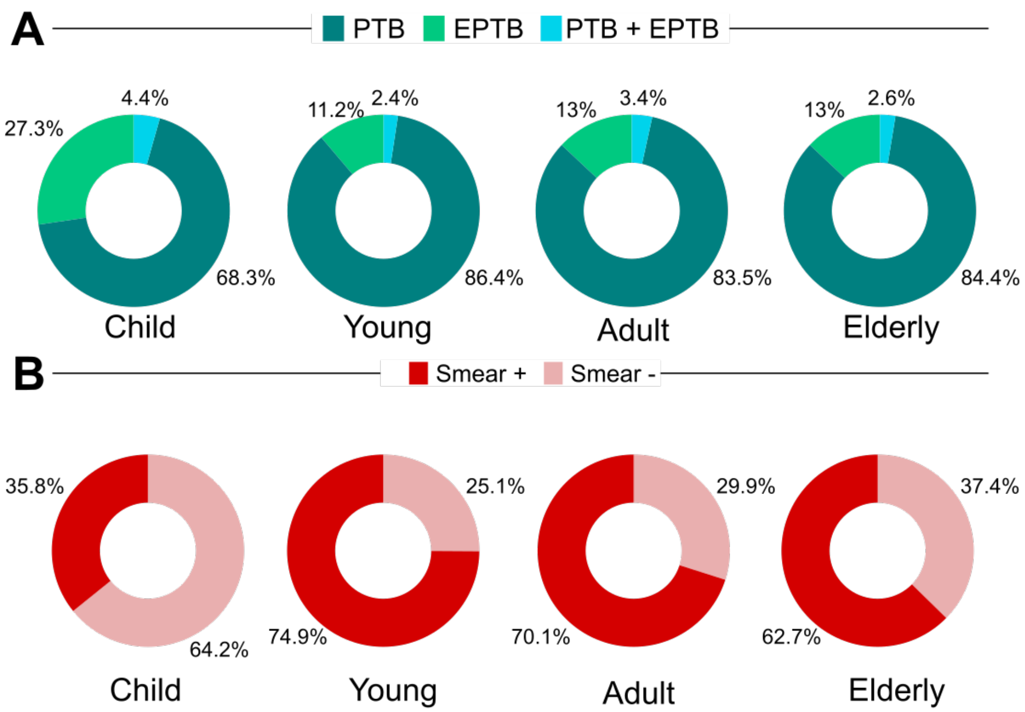

Supplementary Figure 1. Frequency of tuberculosis form (A) and smear results (B) for each age category. Children (0-9 years), Young (10-24 years), Adult (25-64 years), Elderly (≥65 years).

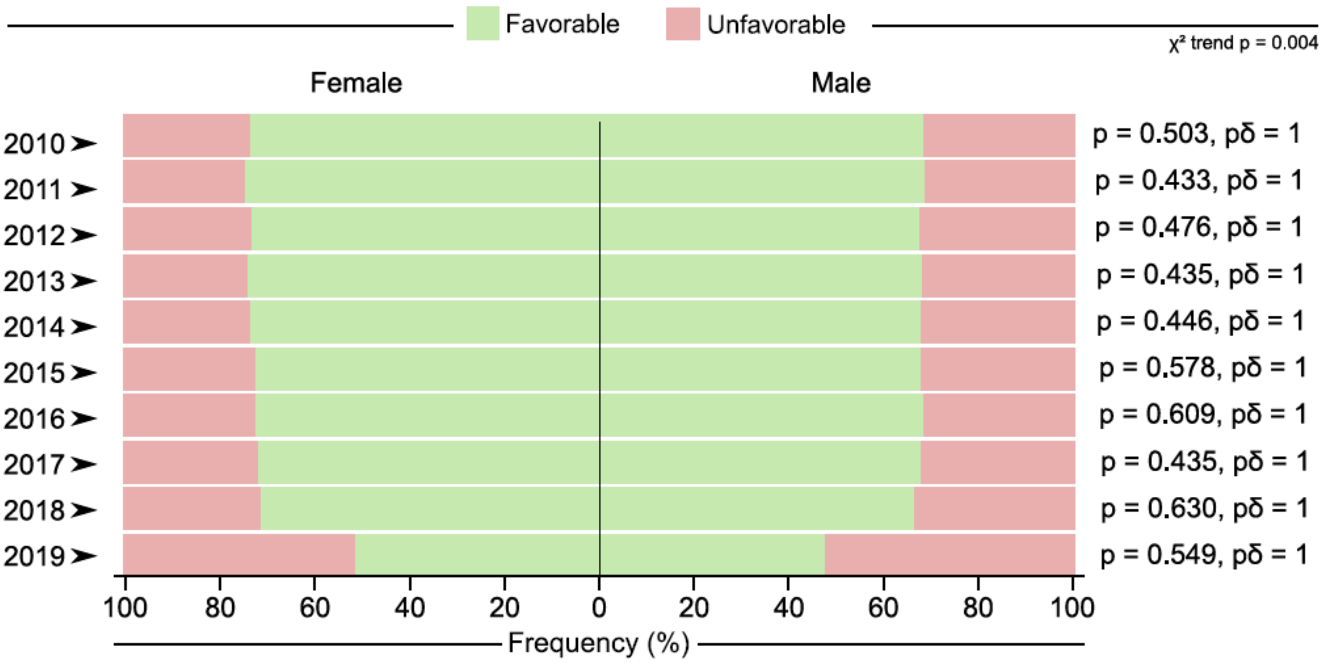

Supplementary Figure 2. Frequency of favorable (green) and unfavorable (pink) outcomes between 2010 and 2019. X^2 trend test was performed to test changes between the years. A Mann-Whitney *U* p-value and second-generation p-value (pδ) were calculated to compare frequencies of outcome between sexes at each indicated year.
